# Observations on Vaccine Inoculation

**Published:** 1804-11-01

**Authors:** 


### *To the Editors of the Medical and Physical Journal.*

GENTLEMEN,

THE observations which have lately appeared in some of the newspapers on the subject of vaccine inoculation, though calculated to excite doubts and fears among the timid and ignorant, yet must fill the minds of those, who with an attentive and impartial eye have watched the rise and progress of this practice, with the greatest indignation.

There is a set of beings in the world so void of just principle, that they constantly oppose whatever is brought forward for the public good. From the first publication of Dr. Jenner's discovery to the present hour, we have seen them ever on the watch, and ready to catch at any event, which might give the smallest pretext for misleading the public in the pursuit of that grand object, the extermination of the small-pox. The late occurrence in Fullwood's Rents has called up one of these malignant spirits, who with an air of exultation tells us, that two children who had had been vaccinated some years ago at the Small-pox Hospital, in St. Pancras, are now supposed to be labouring under the small-pox communicated by contagion. Let us admit the fact, and let us admit too, for it must be admitted, that perhaps there is scarcely a street in the metropolis, or a country town in the British realms, where the same kind of disaster which has happened in Fullwood's Rents may not appear. The reason must be obvious to every perron who will give himself the trouble of reflecting on the subject. The new practice of inoculation has been by many taken up in a hurry, and set on foot without due attention to the rules given for its management. In short, that it has been practised by those who did not understand it, does not admit of dispute. This was fully explained to the Committee of the House of Commons, whose time was occupied several successive weeks in an investigation of the merits of vaccine inoculation, and who reported to the House, their full conviction of its efficacy.—Do you conceive, Gentlemen, that names so celebrated in medical annals as Cline, Home, Farquhar, Baillie, Ash, Blane, Lettsom, Knight, Denman, Ring, Saunders, and many others of the highest character, would have so solemnly born testimony before a committee of their country, in favour of vaccination, if they had not been, by previous experiment, positively certain of its importance? Without similar conviction, would distant nations have made presents to Dr. Jenner, and their universities have heaped upon him their diplomatic honors? There are two ways of doing a thing, the right and the wrong. A person may possess the perfect vaccine matter, such as will produce the guardian pustule; but by managing it injudiciously, or from not knowing all the phenomena of the disease, he fails, and produces one thing for another. There are few practitioners of long standing, and of extensive practice as inoculators of the small-pox, who have not witnessed cases of casual infection after they supposed their patients secure by inoculation. A paper of Dr. Jenner's, published in your Journal for August last, seems to throw much light on this obscure subject; and from the circumstance of the two children in Fullwood's Rents being of the same family, there is great reason to believe that they were under the influence of that disease, which he describes as a frequent impediment to taking either the small-pox or the cow-pox properly.

I repeat again, Gentlemen, that we must be prepared for events similar to those which the newspapers have recorded, corded, while the great work of exterminating that horrid pest the small-pox is going forward. To be convinced that it is going forward, in every part of the world, with a rapidity which its most sanguine promoters could hardly have conceived, we have only to turn our eyes to the continent of Europe and America, and particularly to our settlements in India. That it is not only advancing rapidly but successfully, in many of the largest towns of France, Germany, Italy, Switzerland, &c. &c. we have the most satisfactory proof; and also that the small-pox, which heretofore had committed its destructive ravages among them, is now scarcely known but by name; and while, at home, we are looking upon the Author of this blessing with a cold indifference, he is there the object of enthusiastic regard, and almost idolatrous veneration. In many parts of the world, the Jennerian inoculation was introduced inauspiciously. Prof. Odier of Geneva, began with matter he procured from Vienna, taken from an imperfect pustule. His inoculations were consequently incorrect, and found his patients assailable by the small-pox. But what course did he pursue? Did he, like some of our people at home, decry the practice? No,—he wrote to Dr. Jenner, who furnished him with proper matter and correct instruction, and vaccination went on from that time in so perfect and rapid a manner, that in the large and populous city of Geneva, the small-pox has long since been subdued. For the truth of this statement the reader may refer to German Annals of Medicine or the French. One might go on to any extent, in detailing facts relative to the efficacy of vaccination; but it would be unreasonable to encroach farther on your pages with a subject already so satisfactorily discussed. Yet permit me to make one observation more on the paper in question. That if such writers, instead of indulging their malvolence or prejudice, would exert their understanding, and would give to the subject of vaccine inoculation that share of attention which it deserves, they would at once behold its true and solid importance, and co-operate with those philanthropists whose labors will finally remove one of the greatest impediments which human happiness ever knew.

